# Comparison of the Efficiency of *Banna* Miniature Inbred Pig Somatic Cell Nuclear Transfer among Different Donor Cells

**DOI:** 10.1371/journal.pone.0057728

**Published:** 2013-02-28

**Authors:** Hongjiang Wei, Yubo Qing, Weirong Pan, Hongye Zhao, Honghui Li, Wenmin Cheng, Lu Zhao, Chengsheng Xu, Hong Li, Si Li, Lei Ye, Taiyun Wei, Xiaobing Li, Guowen Fu, Wengui Li, Jige Xin, Yangzhi Zeng

**Affiliations:** 1 Key Laboratory of Banna Miniature Inbred Pig of Yunnan Province, Yunnan Agricultural University, Kunming, China; 2 College of Animal Science and Technology, Yunnan Agricultural University, Kunming, China; University of Nevada School of Medicine, United States of America

## Abstract

Somatic cell nuclear transfer (SCNT) is an important method of breeding quality varieties, expanding groups, and preserving endangered species. However, the viability of SCNT embryos is poor, and the cloned rate of animal production is low in pig. This study aims to investigate the gene function and establish a disease model of *Banna* miniature inbred pig. SCNT with donor cells derived from fetal, newborn, and adult fibroblasts was performed, and the cloning efficiencies among the donor cells were compared. The results showed that the cleavage and blastocyst formation rates did not significantly differ between the reconstructed embryos derived from the fetal (74.3% and 27.4%) and newborn (76.4% and 21.8%) fibroblasts of the *Banna* miniature inbred pig (*P*>0.05). However, both fetal and newborn fibroblast groups showed significantly higher rates than the adult fibroblast group (61.9% and 13.0%; *P*<0.05). The pregnancy rates of the recipients in the fetal and newborn fibroblast groups (60% and 80%, respectively) were higher than those in the adult fibroblast group. Eight, three, and one cloned piglet were obtained from reconstructed embryos of the fetal, newborn, and adult fibroblasts, respectively. Microsatellite analyses results indicated that the genotypes of all cloning piglets were identical to their donor cells and that the genetic homozygosity of the *Banna* miniature inbred pig was higher than those of the recipients. Therefore, the offspring was successfully cloned using the fetal, newborn, and adult fibroblasts of *Banna* miniature inbred pig as donor cells.

## Introduction

*Banna* miniature inbred pigs have been bred since the 1980s from full and half siblings. As unique, highly miniature inbred pigs, *Banna* miniature inbred pigs can serve as large mammalian models with high homozygotic genes and clear genetic background [1], [2]. Given their similar anatomical and physiological features to humans, these animals can be used in various biomedical studies, including disease models, transgenesis, genomics, and xenotransplantation for medical research [3]. Some special traits also appear in inbreeding, such as blindness, deafness, spinal column bend, maxilla defect, and tumor. This particular phenotype provides valuable resources for studying relative human diseases. However, these individuals are hardly reproducible because of their impaired fertility or lethality. Thus, establishing a cloning system is essential to reproduce *Banna* miniature inbred pigs with unique traits for application to studies in various fields.

Somatic cell nuclear transfer (SCNT) is an important method of breeding quality varieties, expanding groups, and preserving endangered species [4]. This method was successfully applied in calf [5], mouse [6], goat [7], pig [8], rabbit [9], cat [10], rat [11], horse [12], mule [13], dog [14], ferret [15], buffalo [16], and camel [17] since the world’s first cloned sheep was obtained in 1996 [18]. Feasible SCNT procedures were established in pig. However, miniature pigs, such as the National Institutes of Health miniature pigs [19] and Clawn miniature pigs, have low cloning efficiency [20]. Under such circumstances, several studies focused on nuclear donor cells, which are generally believed to affect the cloning efficiency in mammals. In cattle, fetal fibroblasts are reportedly more effective than newborn fibroblasts [21]. In pig, fetal fibroblasts are more effective than adult fibroblasts as well as cumulus and oviduct cells [22]. Cell cycle synchronization through differentiation induction enables the effective production of cloned pigs [23]. In mouse, the appropriate combinations of cell type and genotype may improve the efficiency of somatic cell cloning and fetal survival after embryo transfer [24]. However, the cloning process and efficiency in *Banna* miniature inbred pigs remain unclear.

The present study aims to establish the nuclear transfer technology system of *Banna* miniature inbred pig and to investigate the effect of different donor cells, i.e., fetal, newborn, and adult fibroblasts, on the developmental competence of SCNT embryos as well as on the cloning efficiency of this pig.

## Materials and Methods

All animal experiments were performed with the approval of the Animal Care Committee of Yunnan Agricultural University, China.

### Chemicals

Unless otherwise stated, all chemicals were purchased from Sigma Chemical Co. (St. Louis, MO, USA).

### Preparation of Donor Cells

Fetuses (47 days old) isolated from the 22^nd^ generation in the No. 133-family of *Banna* miniature inbred pig were washed three times with phosphate-buffered saline. After removing the head, limbs, and viscera, the fetuses were minced and digested in Dulbecco’s modified Eagle’s medium (DMEM; Gibco) containing 20% fetal bovine serum (FBS; Hyclone), 1% penicillin-streptomycin, and 1 mg/mL Collagenase IV for 4 h at 37°C. The cells were centrifuged at 1000 rpm for 5 min, suspended in DMEM supplemented with 10% FBS and 1% penicillin-streptomycin, and then cultured in a flask until grown to 90% confluence. The cells were then passaged and frozen in DMEM containing 20% FBS and 10% dimethylsulfoxide for future use.

Ear tissues were collected from a newborn piglet of the 18^th^ generation in the No. 111-family and from an adult pig of the 23^rd^ generation in the No. 133-family of *Banna* miniature inbred pig. The fibroblasts were isolated and cultured using the same procedure as described above.

### In vitro Maturation of Oocytes

Porcine ovaries were collected from Hongteng slaughterhouse (Chenggong Ruide Food Co., Ltd, Kunming, Yunnan Province, China) with the permission to use animal parts for this study. The ovaries were transported to the laboratory at 25°C to 30°C in 0.9% (w/v) NaCl solution supplemented with 75 mg/mL potassium penicillin G and 50 mg/mL streptomycin sulfate. Cumulus-oocyte complexes were obtained from follicles 3 mm to 6 mm in diameter using an 18-gauge needle connected to a 10 mL disposable syringe. Cumulus-oocyte complexes with at least three layers of compacted cumulus cells were selected, and approximately 50 oocytes were cultured in 200 µL drops of TCM-199 medium supplemented with 0.1 mg/mL pyruvic acid, 0.1 mg/mL l-cysteine hydrochloride monohydrate, 10 ng/mL epidermal growth factor, 10% (v/v) porcine follicular fluid, 75 mg/mL potassium penicillin G, 50 mg/mL streptomycin sulfate, and 10 IU/mL eCG and hCG (Teikoku Zouki Co., Tokyo, Japan) at 38.5°C in an atmosphere with 5% CO_2_ (100% humidity) (APC-30D, ASTEC, Japan).

### Nuclear Transfer

SCNT was performed as previously described [23], [25]. After culturing for 38 h to 42 h, oocytes with expanded cumulus cells were briefly treated with 0.1% (w/v) hyaluronidase and denuded of cumulus cells using a finely drawn glass capillary pipette. Oocytes extruding the first polar body with uniform cytoplasm were cultured in NCSU23 medium supplemented with 0.1 µg/mL demecolcine, 0.05 M sucrose, and 4 mg/mL bovine serum albumin (BSA) for 0.5 h to 1 h. The oocytes were enucleated by aspirating the first polar body and adjacent cytoplasm using a bevelled pipette (approximately 20 µm in diameter) in Tyrode’s lactate medium supplemented with 10 µM hydroxyethyl piperazineethanesulfonic acid (HEPES), 0.3% (w/v) polyvinylpyrrolidone, and 10% FBS in the presence of 0.1 µg/mL demecolcine and 5 µg/mL cytochalasin B. Any protrusion observed on the surface of an oocyte was removed along with the polar body. Fetal, newborn, and adult fibroblasts of the fourth to ninth passages were used as nuclear donors after cell cycle synchronization by 0.5% FBS serum starvation for 48 h. A single donor cell was inserted into the perivitelline space of an enucleated oocyte.

Donor cells were fused with the recipient cytoplasts with a single direct current pulse of 200 V/mm for 20 µs using an embryonic cell fusion system (ET3, Fujihira Industry Co. Ltd., Tokyo, Japan) in fusion medium [0.25 M d-sorbic alcohol, 0.05 mM Mg(C_2_H_3_O_2_)_2_, 20 mg/mL BSA, and 0.5 mM HEPES (free acid)]. The reconstructed embryos were cultured for 2 h in PZM-3 and then activated with a single pulse of 150 V/mm for 100 µs in an activation medium containing 0.25 M d-sorbic alcohol, 0.01 mM Ca(C_2_H_3_O_2_)_2_, 0.05 mM Mg(C_2_H_3_O_2_)_2_, and 0.1 mg/mL BSA. The reconstructed embryos were equilibrated in PZM-3 supplemented with 5 µg/mL cytochalasin B for 2 h at 38.5°C in humidified atmosphere of 5% CO_2_, 5% O_2_, and 90% N_2_ (APM-30D, ASTEC, Japan).

### Culture of Embryos

Reconstructed embryos were cultured in PZM-3 medium and then placed in an incubator supplied with 5% CO_2_, 5% O_2_, and 90% N_2_ at 38.5°C in a humidified atmosphere. Cleavage and blastocyst formation were monitored on days 2 and 7, respectively. Differential nuclear staining of inner cell mass (ICM) and trophectoderm (TE) cell of the blastocysts was performed. Cell counts were carried out after staining with 10 µg/mL propidium iodide and 10 µg/mL Hoechst33342 under a laser scanning confocal microscope (TCS SP5II, LEICA, Germany) [26], [27].

### Embryo Transfer

Crossbred (Large White/Landrace Duroc) prepubertal gilts weighing 100 kg to 120 kg were used as the surrogate mothers of the cloned embryos. They were checked for estrus at 09∶00 and 18∶00 h daily. Reconstructed embryos cultured for 6 and 30 h after activation were surgically transferred to the oviducts of the estrous surrogate mother by feeding a 14 cm Tom cat catheter (Tyco Healthcare Group LP, MA, USA) through the fimbriae at 0 and 9 h after the first standing estrus was exhibited, respectively. Pregnancy was detected at approximately 23 days after activation using an ultrasound scanner (HS-101V, Honda Electonics Co., Ltd., Yamazuka, Japan).

### Microsatellite Analysis

Parentage analysis was performed in piglets produced by SCNT and the surrogate recipient to confirm the genetic identity of the SCNT piglets with the donor cells. The isolated genomic DNA samples obtained from each newborn piglet (ear tissue) and recipient (ear tissue) were used for microsatellite analysis and sent to a company that specializes in parentage verification for swine (Shanghai GeneCore BioTechnologies Co., Ltd.). Microsatellite analysis of the genomic DNA was performed using 11 porcine-specific microsatellite markers (S0026, S0070, S0155, S0226, SW122, SW24, SW72, SW830, SW840, SW857, and SW936) labeled with the fluorescent dye carboxyfluorescein (FAM).

### Statistical Analysis

For proportional data, the differences between groups were analyzed for variance using the Statview® software package. The level of significance was set at *P*<0.05.

## Results

### Effect of the Donor Cell Type on the Development of Embryos Derived from SCNT

The effect of the donor cell type on the development of embryos derived from SCNT was investigated (Table 1). The cleavage rate and blastocyst formation rate of the reconstructed embryos did not significantly differ between the fetal (74.3% and 27.4%) and newborn (76.4% and 21.8%) fibroblast groups (*P*>0.05), but both groups exhibited significantly higher rates than the adult fibroblast group (61.9 and 13.0%; *P*<0.05). Our results showed that the blastocysts derived from the fetal fibroblasts had more ICM cells, TE cells, total cells and TCM/total cell than those derived from the adult fibroblasts; however, no significant difference was observed among the three groups (*P*>0.05; Table 2).

**Table 1:** Effects of different donor cells on the development of SCNT embryos of *Banna* miniature inbred pig.

Donor cell type	No. of embryos (Repeats)	No. of cleavage (%)	No. of blastocyst (%)
Fetal fibroblast	895(10)	667(74.3±7.7)^a^	254(27.4±5.1)^a^
Newborn fibroblast	843(9)	655(76.4±7.1)^a^	186(21.8±3.5)^a^
Adult fibroblast	1279(10)	780(62.1±7.9)^b^	168(13.5±4.8)^b^

* Values with different superscript letters within a column are significantly different (^a,b^ *P* < 0.05).

**Table 2:** Comparison of ICM and TE cell of blastoysts derived from different donor cells.

Doner cell type	No. of blastocyst	No. of cell			ICM/total cells(%)
		ICM	TE	Total	
Fetal fibroblast	10	10.3±2.6^a^	36.5±5.6^a^	46.8±7.2^a^	21.8±4.3^a^
Newborn fibroblast	10	8.5±2.0^a^	33.4±6.6^a^	41.9±8.0^a^	20.8±3.0^a^
Adult fibroblast	10	6.9±1.7^a^	31.0±5.3^a^	37.9±6.5^a^	18.2±2.6^a^

* Values with same superscript letters within a column are not significantly different (^a^ *P*>0.05).

### Effect of the Donor Cell Type on the Implantation Rate of Embryos Derived from SCNT

**Figure 1:**
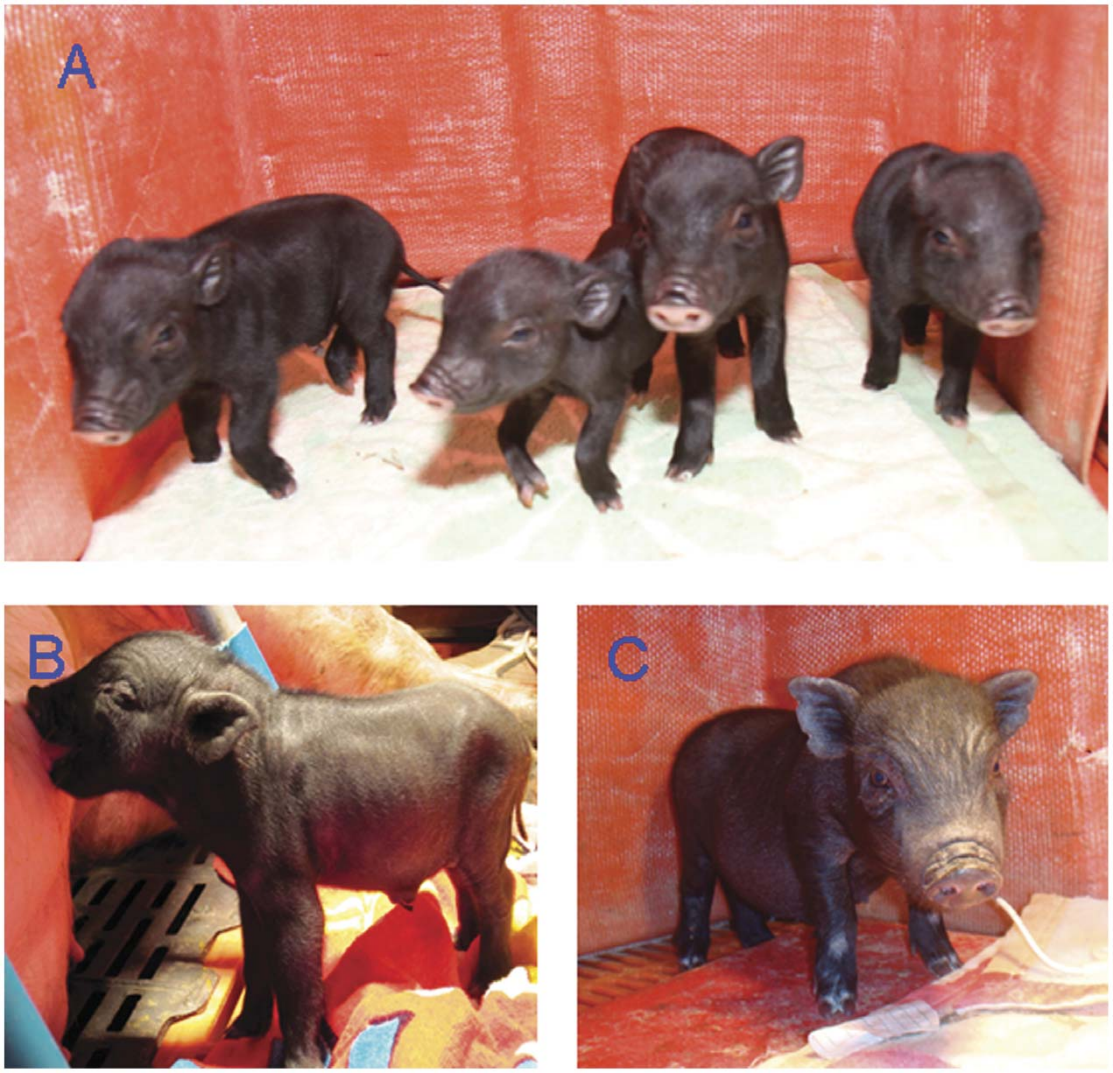
*Banna* miniature inbred pigs cloned by SCNT. Piglets derived from (A) fetal fibroblasts, (B) newborn fibroblasts, and (C) adult fibroblasts.

Reconstructed embryos derived from fetal, newborn, and adult fibroblasts were transferred to five, five, and three surrogate mothers, respectively. For the fetal, newborn, and adult fibroblasts, the number of pregnancies were three (60.0%), four (80.0%), and one (33.3%), respectively, and the number of deliveries were three (60.0%), one (20.0%), and one (33.3%), respectively (Table 3). Eight, three, and one cloned piglets were obtained from the fetal, newborn, and adult fibroblasts (Figure 1). Two of three neonatuses from the newborn fibroblasts died shortly after birth because of neonatal asphyxia caused by dystocia. In the newborn fibroblast group, the uteri of two surrogate mothers were dissected on pregnancy day 120, and 20 and 13 absorbed fetuses were collected, which took on a lump without the shape of a normal fetus. Another surrogate mother was midway aborted. The birth weight of the piglets derived from the fetal, newborn, and adult fibroblasts were 817.8, 741.3, and 925.0 g, respectively (Table 4); no significant difference in body weight was observed among the three groups (*P*>0.01). These weights of the fetal, newborn, and adult fibroblast groups were significantly greater than that of the control groups (*P*<0.01).

**Table 3:** Development of cloned embryos derived from different donor cells after being transferred to surrogate gilts.

Donor cell type	No. of surrogates	No. of transferred embryos	No. of pregnancy (%)	No. of delivery (%)	Offspring (dead)
Fetal fibroblast	5	246.0±65.7	3(60.0%)^a^	3(60.0%)^a^	8
Newborn fibroblasts	5	148.0±40.3	4(80.0%)^a^	1(20.0%)^a^	3(2)
Adult fibroblast	3	304.7±20.0	1(33.3%)^a^	1(33.3%)^a^	1

* Values with same superscript letters within a column are not significantly different (^a^ *P*>0.05).

**Table 4:** Comparison of birth weight of cloned piglets derived from different donor cells.

Donor cell type	Fetal fibroblast (♂)	Newborn fibroblast (♂)	Adult fibroblast (♀)	Control	
				♂	♀
No. of piglets	8	3	1	20	20
Birth weight(g)	817.8±157.1^a^	741.3±156.0^a^	925^a^	518.3±114.4^b^	503.6±110.4^b^

* Values with different superscript letters within a column are significantly different (^a,b^ *P* < 0.01).

### DNA Parentage Analysis

Parentage analysis was performed on the cloned piglets, donor cells, and surrogate females. The genotype of each piglet from fetal, newborn, and adult fibroblasts was identical to the donor cell but different from its surrogate mother. Only two, one, and two heterozygous loci were observed in the 11 porcine-specific microsatellite markers of the donor cell line from the fetal, newborn, and adult fibroblasts, respectively. The gene homozygosity of all donor cells was higher than that of the surrogate mothers (Tables 5, 6, 7).

**Table 5:** Microsatellite analysis of cloned piglets derived from fetal fibroblasts.

Marker	Dye name	PCR annealing temp	Genotypes of Recipient			Cell line genotypes (BN133)	Genotypes of litter							
			1	2	3		1	2	3	4	5	6	7	8
S0026	FAM	55	97	93/101	91/97	97	97	97	97	97	97	97	97	97
S0070	FAM	55	271/273	285/287	263	271/273	271/273	271/273	271/273	271/273	271/273	271/273	271/273	271/273
S0155	FAM	55	142/152	142/152	152/156	142	142	142	142	142	142	142	142	142
S0226	FAM	55	183/195	193	183	195	195	195	195	195	195	195	195	195
SW122	FAM	55	108	108/110	106/110	108	108	108	108	108	108	108	108	108
SW24	FAM	55	103	101/103	115	103	103	103	103	103	103	103	103	103
SW72	FAM	55	97	99/109	97	97	97	97	97	97	97	97	97	97
SW830	FAM	50	180/184	168	180	180/184	180/184	180/184	180/184	180/184	180/184	180/184	180/184	180/184
SW840	FAM	55	135	117/123	125/129	135	135	135	135	135	135	135	135	135
SW857	FAM	55	139/155	139/151	143/153	139	139	139	139	139	139	139	139	139
SW936	FAM	55	97	95/109	93/119	97	97	97	97	97	97	97	97	97

* For each microsatellite marker, genotype was determined by size (base pairs). Two numbers for each sample at each locus represent the PCR product size at that particular locus.

* Litters 1,2,3 came from recipient 1; litters 4,5,6 came from recipient 2; and the rest came from recipient 3.

**Table 6:** Microsatellite analysis of cloned piglets derived from newborn fibroblasts.

Marker	Dye name	PCR annealing temp	Genotypes of Recipient	Cell line genotypes	Genotypes of litter		
					1	2	3
S0026	FAM	55	97	93	93	93	93
S0070	FAM	55	273/283	273	273	273	273
S0155	FAM	55	152/158	142	142	142	142
S0226	FAM	55	181	195	195	195	195
SW122	FAM	55	112	108	108	108	108
SW24	FAM	55	103	115	115	115	115
SW72	FAM	55	106/108	98/110	98/110	98/110	98/110
SW830	FAM	50	177	185	185	185	185
SW840	FAM	55	125	125	125	125	125
SW857	FAM	55	144	154	154	154	154
SW936	FAM	55	109	97	97	97	97

**Table 7:** Microsatellite analysis of cloned piglets derived from adult fibroblasts.

Marker	Dye name	PCR annealing temp	Genotypes of Recipient	Cell line genotypes	Genotypes of litter
S0026	FAM	55	92/96	96	96
S0070	FAM	55	263/271	271	271
S0155	FAM	55	146/158	142	142
S0226	FAM	55	180/198	192	192
SW122	FAM	55	110/118	108	108
SW24	FAM	55	115	103	103
SW72	FAM	55	98/106	97	97
SW830	FAM	50	180	180/185	180/185
SW840	FAM	55	125	125/135	125/135
SW857	FAM	55	152	152	152
SW936	FAM	55	108	98	98

## Discussion

This study is the first to report on successful cloning using the fetal, newborn, and adult fibroblasts of *Banna* miniature inbred pig. This pig can be extensively used as a large animal model in biomedical research. It can also be used a source of organs for xenotransplantation in humans because of its highly homozygotic genes and clear genetic background.

However, low cloning efficiency has hampered the production of cloned animals. Several reports indicated that the type of donor cell can affect the birth rate. In mouse, an appropriate interaction between cell type and genotype can improve cloning efficiency [24], [28]. In cattle, clones derived from adult cells aborted in the later stages of pregnancy and calves developing to term show a higher number of abnormalities than those derived from newborn or fetal cells [29]. The simultaneous coordination of the donor cell type and cell cycle stage can maximize the overall cloning efficiency [30]. However, in buffalos, cumulus cells are a more efficient nuclear donor for SCNT than skin fibroblast and granulosa cell lines [31]. Sheep also shows breed-specific variability in terms of cloned embryo development [32]. Nevertheless, neither the donor cell type nor the gender significantly affects the overall efficiency of the in vitro production of SCNT sheep embryo [33]. In rabbit, embryos reconstructed with fresh cumulus cells have a more efficient developmental potential than those reconstructed with fetal fibroblasts in vivo and in vitro [34]. In pig, the type of donor somatic cell is important for the development of cloned embryos; fetal fibroblasts are the most effective among adult and fetal fibroblasts, cumulus and oviduct cells [22]. However, comparisons show that adult cells of any type are inferior to fetal fibroblasts in terms of reconstructed embryo development.

Our results reconfirmed the fact that the type of donor somatic cell is critical for determining developmental competence. Moreover, these results further confirmed that fetal fibroblasts have the highest efficiency as donor cells in SCNT for the cloning of highly inbred *Banna* miniature pigs in three types of donor fibroblast, whereas adult fibroblasts have the least efficiency. The fetal fibroblasts of *Banna* miniature inbred pig have a cloning efficiency of 0.65%, which is higher than that (0.4%) of newborn fibroblasts. However, these cloning efficiencies were both higher than that (0.1%) of adult fibroblasts. The very low efficiency of adult fibroblasts as donor cells in SCNT could be attributed to the very low cleavage rate and blastocyst formation rate as well as to the very low numbers of ICM, TE and total cells in the blastocysts. In addition, compared with the fetal and newborn fibroblasts in the primary culture, adult fibroblasts showed slightly slower proliferation rate (data not shown). Previous reports demonstrated that the developmental rates of cloned embryos remain similar regardless of the donor age in several other different species [35]–[37]. However, significant differences in developmental rate and birth rate exist among the donor cells of different ages in pigs [22]. The possible reason for the decreased potential of fibroblasts as donor cells in producing cloned healthy live birth with increasing age may also be attributed to the differentiation status of donor cells. Fetal cells are highly undifferentiated and more amenable to reprogramming after reconstruction than differentiated cells [22], [38]. Furthermore, the somatic cells of adult animals accumulate more genetic aberrations and are more terminally differentiated than fetal cells [39], [40]. Thus, somatic cells are more likely to fail at full-term development with increasing age.

Several studies reported SCNT attempts in miniature pig. The donor cells of these pigs are all derived only from fetal fibroblasts. The cloning efficiency of the Chinese *Bama* miniature pig is 0.11% [1/870 (no. offspring/no. embryos transferred in the recipients; similarly hereinafter)] [41]. The efficiencies of producing cloned Potbelly miniature pigs from the lung and kidney of male newborn Meishan pigs as recipients are 8.57% and 3.57% (3/35 and 2/56), respectively [42]. Both the reconstructed embryos of *Bama* and Potbelly miniature inbred pigs were transferred at the two- to four-cell stage. Clawn miniature pig developed from cloned embryos was transferred to recipients after culturing for 6 h to 40 h and showed a cloning efficiency of 2.35% (2/85) [20]. Yucatan miniature pig was successfully cloned at an efficiency of 1.1% (7/631). Embryos were cultured for less than 1 h before being surgically transferred into the recipient [43]. The production efficiencies of cloned Nippon Institute for Biological Sciences strain miniature pigs using male and female fetal fibroblasts as nucleus donors range from 0.64% (2/314) to 0.9% (3/331) by transferring reconstructed embryos cultured for 1 day to 2 days into miniature and common domestic pigs [44]. In the National Institute of Health miniature inbred pig, the cloning efficiency is 1.3% (21/1610) using fetal fibroblasts as donor cells and transferring on the same day of SCNT [19]. In several inbred mice that have not been cloned (such as C57BL/6 and C3H/He), the cloning efficiencies of cloned inbred strains are extremely low, similar to the DBA/2 and 129/Sv strains [24], [37]. For the first time that *Banna* miniature inbred pigs were cloned using fetal fibroblasts, the cloning efficiency is 0.65% for transferring at the one- to two-cell stage. The cloning efficiency of the miniature pig is relatively low compared with others, which may be ascribed to the inbred genetic background and incorrect epigenetic modification resulting from imperfect genomic reprogramming [19], [45], [46]. Previous studies have reported that SCNT-derived clones are prone to various abnormal phenotypes, including large birth weight [47], [48]. Our result showed that the birth weight of cloned *Banna* miniature piglets is much larger than that of non-cloned *Banna* miniature inbred pigs. This finding can be attributed to the different body sizes of the surrogate mothers. In this study, all reconstructed embryos were transferred into crossbred Large White/Landrace Duroc surrogated mothers. The body size of the surrogated mother for the cloned *Banna* miniature piglet was approximately 200 kg, which is larger than that of *Banna* miniature inbred sow (approximately 50 kg). Although the uterus of the surrogate mother is larger than that of the *Banna* miniature inbred sow, it carries fewer cloned fetuses than the *Banna* miniature inbred sow. Thus, the cloned *Banna* miniature piglet fetuses can obtain more nutrition and large developmental space from large surrogate mothers than non-cloned *Banna* miniature inbred pigs from *Banna* miniature inbred sow. As a result, cloned *Banna* miniature inbred piglets have significantly larger birth weights than non-cloned *Banna* miniature inbred pigs. DNA parentage was performed, the genotype of each litter was identical to its donor cell but different from its surrogate mother. As an inbred line, the homozygosis of the donor cell genotype of the *Banna* miniature pig is higher than that of the others.

In conclusion, *Banna* miniature inbred pig offspring was successfully cloned using the fetal, newborn, and adult fibroblasts of this animal as donor cells. The cloning efficiency of the fetal fibroblasts was significantly higher than those of the other two fibroblasts. In addition to the establishment of a cloning system, physiological and reproductive studies on cloned *Banna* miniature inbred pig are required. The results will benefit animal models, transgenesis, genomics, and xenotransplantation.
